# Monozygotic dichorionic-diamniotic twin pregnancy after single embryo transfer at blastocyst stage: a case report

**DOI:** 10.5935/1518-0557.20200052

**Published:** 2021

**Authors:** Samia I Jundi, Nikolas C de A Pereira, Thaís M Merighi, Jéssica F dos Santos, Isaac M Yadid, Márcio Coslovsky, Thelma S. Criscuolo, Ivan A de A Penna

**Affiliations:** 1 Faculdade de Medicina da Universidade Federal Fluminense. Niterói, RJ, Brazil; 2 Clínica Primordia Medicina Reprodutiva. Rio de Janeiro, RJ, Brazil; 3 Clínica Fert Rio. Rio de Janeiro, RJ, Brazil

**Keywords:** ICSI, SET, dichorionic, diamniotic, monozygotic twin

## Abstract

Single embryo transfer is highly encouraged on *in vitro* fertilization due to its lower rates of multiple pregnancy. Nevertheless, the likelihood of multiple pregnancy is higher when using assisted reproductive technology, probably because of embryo handling. Timing is crucial in the post-fertilization division of a single embryo to establish the amniocity and chorionicity of the gestation. In the case reported a 38 year-old woman, nulligravid, had a single blastocyst implanted, which resulted in monozygotic dichorionic-diamniotic twins. Despite being rare, there are reports of similar cases questioning the current knowledge on time of embryo division and the impact of assisted reproduction.

## INTRODUCTION

Assisted reproduction technology (ART) enabled many infertile couples to fulfill their desire to become parents. Consequently, to using these techniques, there was an increase in twin pregnancies, mainly dizygotic, due to the implantation of more than one embryo. However, there is also the rare occurrence of monozygotic twins (MZT), by mechanisms that we still do not understand fully (Knopman *et al*., 2014; Sundaram *et al*., 2018; Vitthala *et al*., 2009). In general, the prognosis of a multiple pregnancy presents worse maternal and neonatal outcomes; thus, it is essential to determine and identify the risk factors to create strategies and reduce the occurrence of these events (Knopman *et al*., 2014).

The incidence of monozygotic twins in natural pregnancies is approximately 0.4%, this rate after *in vitro* fertilization (IVF) ranges from 0.2 to 12%. However, the least commonly found event after IVF is the occurrence of MZT multifetal dichorionic-diamniotic (DC-DA) gestation, with a rate ranging from 0.04 to 5.5% (Sundaram *et al*., 2018; Vitthala *et al*., 2009). This case report is about another MZT DC-DA pregnancy after a single blastocyst transfer.

## CASE REPORT

A 38-year-old female, nulligravid, and her 39-year-old husband came to us to investigate their infertility condition. She stopped using combined oral contraceptives five years ago, performed timed intercourse twice four years ago without therapeutic success and three years ago performed infertility investigation. Their test results showed: anti-Mullerian hormone: 1 ng/mL; 7 antral follicle count in the ultrasound; and an endometrial polyp in her video hysteroscopy. The spermogram was normal as for the WHO parameters. Hysterosalpingography was positive with cotte bilaterally, but with fixed tubes. Another semen analysis performed showed 2% Kruger morphology. The hormonal tests performed by both were normal. Therefore, we concluded that their infertility condition had male factor and tubal factor. We decide to perform intracytoplasmic sperm injection (ICSI).

We performed controlled ovarian stimulation, for FIV, with 1950 UI of follitropin alfa/lutroprin alfa (Pergoveris^®^) and 1050 UI of menopause gonadotropin (Merional^®^) during 10 days, and we administered GnRH-antagonist (Cetrotide^®^) from the 6th day to the trigger. On day 11, there were 10 follicles, ranging from nine to 23mm in diameter. On day 11, we injected 250µg of choriogonadotropin (Ovidrel^®)^. After 36 hours, we collected eight eggs, five of them were mature (MII) and injected.

Five mature oocytes were fertilized by ICSI and after 130 hours one of them reached blastocyst stage, morphologically classified as B3AA, according to the classification adapted from Gardner et al. (2000) for the protocols of the FIV Primordia lab, on day 5. We vitrified the embryos in CRYOTOP^®^ with vitrification medium, both from Kitazato. We also used the Kitazato medium for thawing , following the manufacturer’s instructions and standard technique of the Primordia laboratory.

**Figure 1:**
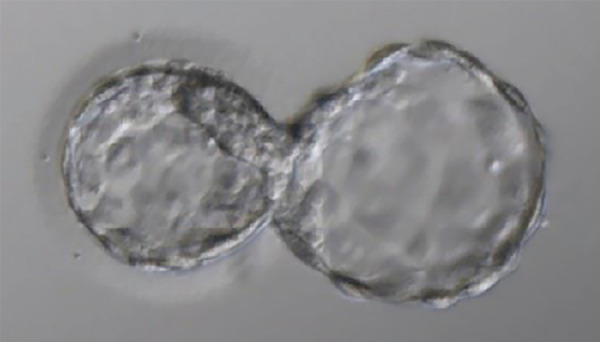
Embryo expansion after hatching in an image obtained using an optical microscope just before embryo transfer

**Figure 2:**
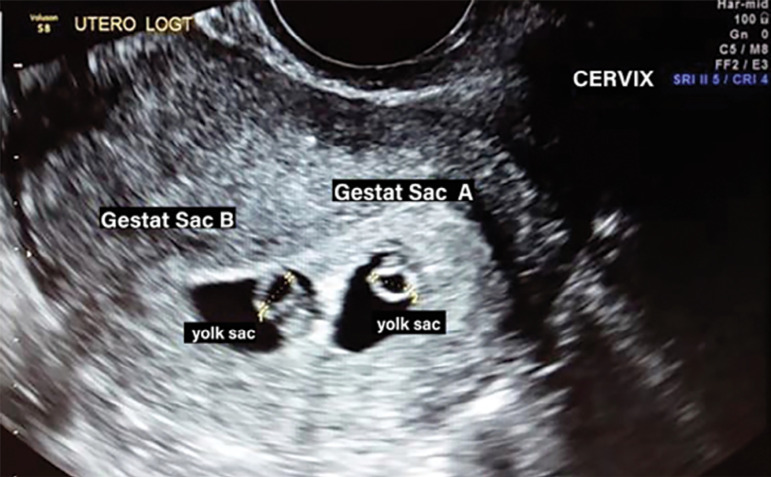
USG performed 5 weeks after single embryo transfer, showing two gestational sacs

After thawing, we checked the surviving embryo in an inverted microscope; performed 1/3 assisted hatching of the blastocyst and maintained it in culture for 4 hours. The embryo transfer occurred guided by ultrasound and using a sterile embryo transfer catheter (Guardia TM-handle), in the estrogen plus progesterone cycle (Figure 1). We scheduled the single embryo transfer (SET) when the ultrasound demonstrated a trilaminar endometrium measuring 8.5 mm and no follicles in both ovaries. The transfer was uneventful and there was no blood in the catheter. We had a positive pregnancy test (209,4 mUI/mL) 10 days after the transfer. Five weeks later we performed an ultrasound scan, which showed two gestational sacs (Figure 2).

## DISCUSSION

Whenever possible, recommendations today are for a single embryo transfer (SET), considering that multiple pregnancies have higher rates of maternal and neonatal morbidity and mortality (McLernon *et al*., 2010). However, even following the guidelines and choosing SET, there is still the possibility of multiple monozygotic pregnancies by embryo splitting and dizygotic pregnancy (Ikemoto *et al*., 2018).

Our case is another report of conjugal infertility treated with ICSI and SET that resulted in MZT, DC-DA pregnancy. When we performed the transfer, the couple had no sexual intercourse and the USG before embryo transfer showed no follicles, allowing us to discard a dizygotic pregnancy.

Some of the known risk factors associated with MZT pregnancy after SET are: mother's age < 35y, blastocyst transfer, ICSI, assisted hatching and freeze-warming cycles, while there has been no difference concerning ovarian stimulation method (Ikemoto *et al*., 2018; Busnelli *et al*., 2019). Mechanisms that may explain this phenomenon are being studied, such as, for example, blastomere herniation hypothesis, depressed calcium levels in the early embryo, delayed implantation, cell repulsion theory, cell-to-cell adhesion impairment, and the existence of a codominant axis (Knopman *et al*., 2014).

A systematic review published by Busnelli *et al.* (2019) analyzed 40 studies and reported that blastocyst transfer and female age <35 years were associated with a statistically significant increase in MZT rates after IVF. When they compared conventional IVF with ICSI and assisted hatching, they were associated with an increased risk of MZT pregnancy; while embryo biopsy, cryopreservation, and oocytes donation were not (Busnelli *et al*., 2019). In a Japanese retrospective study published by Ikemoto *et al.* (2018) with almost 1 million single embryonic transfer cycles, the prevalence of multiple pregnancies with zygotic splitting was 1.36% of the 276 934 clinical pregnancies after SET. Frozen-warmed embryonic transfer cycles, blastocyst culture, assisted hatching and cycles with cleavage embryos had greater odds of MZT (Ikemoto *et al.*, 2018).

There are controversies concerning when after fertilization a single embryo splits in two to form a dichorionic-diamniotic (DC-DA) pregnancy. Classical dogma states that if the embryo divides in the first 3 days post-fertilization, it results in DC-DA pregnancy, embryo division between days 4 and 8 post-fertilization produces monochorionic-diamniotic (MC-DA) twins and division between 9 and 12 days post-fertilization results in monochorionic-monoamniotic (MC-MA) pregnancy. However, in case-report studies the majority of DC-DA occurred at the blastocyst stage (Sundaram *et al*., 2018; Kyono, 2013).

Of the aforementioned risk factors, our case presents ICSI fertilization, frozen-warmed cycles, blastocyst transfer and assisted hatching. In accordance with other reports, we present another DC-DA MZT transferred at the blastocyst stage. The mechanisms behind these events are still unclear, with some theories reported by Busnelli *et al*. (2019). Since it is a rare event, case reports are fundamental to enlarge the sample, enable better associations between risk factors, and improve our understanding of the underlying mechanisms behind MZT DC-DA pregnancies.
